# Anxiety and depression in natural caregivers of patients with schizophrenia

**DOI:** 10.1192/j.eurpsy.2023.2239

**Published:** 2023-07-19

**Authors:** F. Znaidi, S. Ellouze, R. Ghachem

**Affiliations:** 1 Department Of Psychiatry, Razi Hospital of Manouba, Manouba; 2 Department Of Psychiatry, Hedi Chaker University Hospital, Sfax, Tunisia

## Abstract

**Introduction:**

Natural caregivers of patients with schizophrenia constitute a main source of care as they have to shoulder a multitude of caregiving responsibilities and are then confronted with considerable difficulties while providing care. As a result, natural caregivers, often described as “the hidden patients” usually suffer from psychological consequences such as anxiety and depression.

**Objectives:**

This study aimed to asses anxiety and depression among natural caregivers of patients with schizophrenia and to identify risk factors for developing such disorders.

**Methods:**

We conducted a cross-sectional, descriptive and analytical study, including 80 natural caregivers of patients with schizophrenia. We used the Hospital Anxiety and Depression Scale (HADS) to evaluate anxiety and depression.

**Results:**

The typical caregiver profile was consistent with a 55-year old married illiterate first degree relative (mostly parents or spouses) with a low socio-economic level.

The mean anxiety score was 10.6± 5,1 and the mean depression score was 11.6± 6.2. Depression and anxiety were diagnosed in 66% of caregivers.

Anxiety and depression scores were significantly higher among female illiterate unemployed caregivers, those with organic history and among parents and correlated with the caregiving duration.

Anxiety scores were higher when patients in charge had poor therapeutic adherence and aggressive behavior and correlated with the age of caregivers and the number of other sick persons in charge.

Caregivers reported higher levels of depression when patients in charge were not married, unemployed and had a history of suicide attempts.

Anxiety score were significantly correlated with depression scores.

**Conclusions:**

Caregivers of patients with schizophrenia, although thought to be a privileged source of emotional and social support, are hardly taken into account following the deinstitutionalization movement.

**Disclosure of Interest:**

None Declared

